# Investigate-Design-Practice-Reflect: An Iterative Community-Engaged Action Process to Improve Population Health

**DOI:** 10.1007/s10900-024-01385-y

**Published:** 2024-08-07

**Authors:** Marisa S. Rosen, Ann E. Rogers, Mary J. Von Seggern, Brandon L. Grimm, Athena K. Ramos, Michaela A. Schenkelberg, Regina E. Idoate, David A. Dzewaltowski

**Affiliations:** 1 College of Public Health, Department of Health Promotion, University of Nebraska Medical Center, Omaha, NE USA; 2 College of Education, Health, and Human Sciences, School of Health and Kinesiology, University of Nebraska at Omaha, Omaha, NE USA

**Keywords:** Community Engagement, Cross-sector Collaboration, Quality Improvement, Community Action, Coalitions

## Abstract

**Background:**

Community-based coalitions are a common strategy for community engagement efforts targeting the improvement of a variety of population health outcomes. The typical processes that coalitions follow to organize efforts include steps that are sequential, slow, and time intensive. These processes also limit local decision-making to the selection of evidence-based policies or programs.

**Methods:**

We present a process control theory-based *Community Action Process*, Investigate-Design-Practice-Reflect (IDPR), where community hubs (i.e., coalitions) organize agile efforts in a non-sequential, rapid, and efficient manner to harness local assets and data to make decisions regarding the provision and production of population health services. Using qualitative methods, we illustrate and analyze the use of IDPR in a one community case study as part of Wellscapes, a Type 3-hybrid implementation-effectiveness community randomized controlled trial to improve children’s population health physical activity.

**Results:**

We found community members followed the IDPR *Community Action Process* to rapidly design, organize, deliver, and receive feedback on a community-based, children’s population physical activity prototype, an afterschool Play-in-the-Park opportunity for all children.

**Discussion:**

Following IDPR afforded the community coalition timely learning through feedback within a process that coordinated decisions regarding what community services met community needs (provision decisions) and how to organize the production of the population health services (production decisions).

## Introduction

Since the 1990s, Community Engagement (CE), defined as “the process of working collaboratively with groups of people who are affiliated by geographic proximity, special interests, or similar situations with respect to issues affecting their well-being" [1–3], has become standard practice to improve population health, or the health outcomes of a ‘group’ and the distribution of outcomes within the ‘group’ [4]. In health care, the ‘group’ is a patient population or all people in the healthcare system, while in public health, the ‘group’ is often defined as all people living in a geographic area [5]. CE strategies often include forming small groups of stakeholders, frequently called an advisory committee or coalition, and center around “developing relationships that enable stakeholders to work together to address health-related issues and promote well-being to achieve positive health impact and outcomes” [6]. However, an array of CE models, frameworks, and processes define the steps to facilitate stakeholders to work together to improve population health outcomes [7].

One overarching way to categorize CE approaches is along a continuum of increasing levels of community involvement: outreach, consult, involve, shared leadership/participatory, and community-driven [1, 8]. While a shared leadership and participatory process shares control both within and outside of a local community, a community-driven process gives control of all decisions and resources to local community groups [9]. Thus, the World Health Organization (WHO) views CE not as a collaboration process between outsiders, such as researchers and community members, but rather as a capacity-building process where outsiders facilitate the development of relationships between stakeholders to achieve population health improvement [10].

**Fig. 1:**
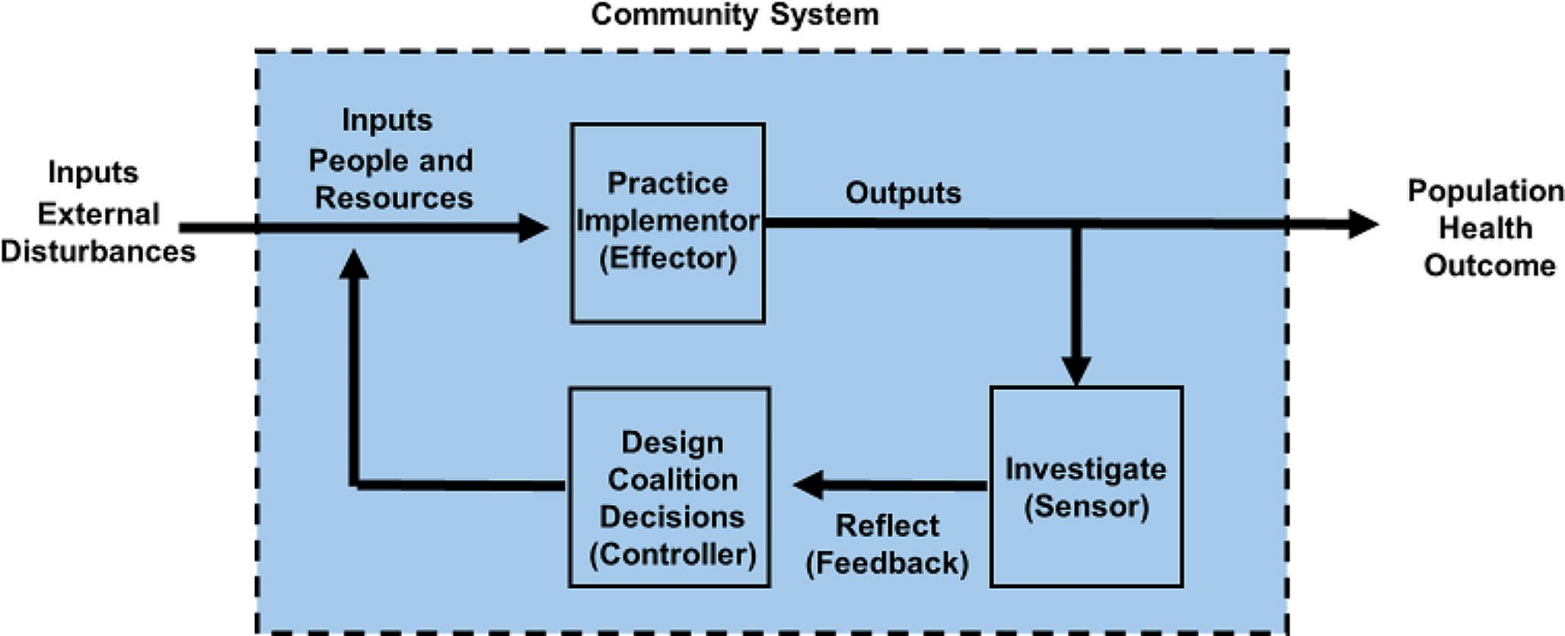
Whole-of-community process control system

To inform community-driven processes, we can turn to fundamental principles of control systems theory (CST), which dictates that individual and collective decision-making processes and actions are informed by a feedback control system comprised of three essential functions: sensor, controller, and effector (Fig. 1) [11–13]. The role of the sensor is to monitor and collect information on the system to inform a judgment of the state of the system. The controller is a decision-making unit that compares the state of the system with a desired state (often defined in business and health care as a quality reference standard) and makes decisions about how to organize inputs (e.g., people and resources) into a process (e.g., set of activities) that produces outputs, which are designed to effect or move the system toward the desired state (e.g., improved population health). The effector(s) or implementor(s) follows a process of activities decided on by the controller to produce an output. When combined, the sensor, controller, and effector operate collectively to provide the architecture for a feedback loop, which provides information for improved decision-making on what to provide (provision decision) and how to provide (production decision) outputs that move the system closer to the desired state of the quality reference standard and/or population health outcome.

Within healthcare system population health improvement, where the group is a patient population, feedback control system functions have been applied to system improvement through Plan, Do, Study, Act (PDSA) cycles [14, 15]. The PDSA cycle has roots in both Shewhart’s 1930 work, where he defined the production of a product as a problem of control [16], and in Deming’s later application of Shewhart’s work, which refined and popularized the production of products and services as a control problem that was solved using a control loop [17]. Building upon the use of PDSA cycles, Langley and colleagues created the Model for Improvement [18] and Berwick popularized this system improvement strategy as an iterative, continuous improvement process as follows: First, a group of within-organization stakeholders form a team that asks three questions: (1) What are we trying to accomplish, (2) How will we know that a change is an improvement, and (3) What change can we make that will result in an improvement, followed by the PDSA process. From a control systems theory perspective, the questions define how to design the feedback control system functions, including a *sensor* to measure the improvement and the team to serve as a *controller* to make decisions or plan what will be done and how (PLAN). Next, the decisions are implemented to be an *effector* on the system (DO). Third, the *feedback* of sensed change is then compared to standards (STUDY) to control the system through future feedback control decisions. Finally, several rapid learning control loops are implemented to learn the specifications for spreading the defined process on a larger scale (ACT) [14, 15, 18].

For community system improvement, Shewhart and Deming’s work was used to design a different “model for improvement” called the Collective Impact model (CI) [19]. CI is a process that organizes work across sectors to bring together stakeholders from different organizations across a community in a coalition to control or coordinate action through collective planning, implementation of activities, and feedback on accountability to the plan to improve a population health outcome. In its original formulation, CI, applied CST in a business quality improvement model, aimed to foster the “commitment of a group of important actors from different sectors to create a common agenda for solving a specific problem,” thus building the group’s capacity [19]. Using feedback control system functions, we can label the components of CI as follows: shared measurement system (sensor), group of stakeholders committed to a common agenda (controller), mutually reinforcing activities (effector), and continuous communication supported by backbone organization (feedback).

Despite efforts to improve population health outcomes through the integration of control systems theory into community systems, gaps remain. In a systematic review of whole community interventions targeting preventive population health behaviors for cancer risk, our team developed a typology to identify the presence of a sensor (e.g., data with continuous feedback for community change), a controller of a coalition following a CE process (e.g., continuum of CE ending with local control over design of the intervention), and an effector (e.g., continuum of CE ending with local community members implementing the intervention) [20]. Using this typology, we found that few community-based interventions included the fundamental functions necessary for community system coordination, particularly where the CE process is part of a solution to a community coordination system problem [20].

Additionally, in subsequent studies, our research team has found that investigating the existing community of people and resources and organization of the elements into a production system, such as decision-makers controlling resource allocation and use of existing assets to produce services, is a critical first step in coordinating system improvement, which is often lacking in the current system improvement literature [21–24]. To fill this gap, our team developed Investigate-Design-Practice-Reflect (IDPR), a continuous, rapid-cycle, quality improvement process to coordinate a system to improve population health outcomes. IDPR follows the functions of fundamental feedback process control system: I (Sensor), D (Controller), P (Effector), R (Feedback) (Table 1).

**Table 1:** Investigate-Design-Practice-Reflect (IDPR) Process

IDPR Phase	Control System Construct & Definition	Overarching Question
Investigate	Sensor: Detects and measures some effect and feeds back the information into the system.	What is the boundary of the system? What is the structure and function of local elements?
Design	Controller: Provides decisions and control commands.	What are potential solutions?
Practice	Effector: Performs control commands.	What can be implemented now?
Reflect	Sensor Feedback: Provides insights into the system and pushes the system away from homeostasis.	What was successful, and what needs to change?

The primary objective of this paper is to describe the development and components of the IDPR *Community Action Process*. To describe IDPR, we use the Wellscapes project, to provide an example of how IDPR can be adapted to guide a community to improve a population health outcome, such as childhood physical activity (PA).

## Materials and Methods

### Setting and Participants

The Wellscapes Initiative (www.wellscapes.org) is a two-wave staggered-start community development initiative to improve PA of 3rd through 6th-grade children in rural Nebraska. As part of a Type 3-hybrid implementation-effectiveness community randomized controlled trial (ClinicalTrials.gov Identifier: NCT03380143), the research team worked with representatives from local public health departments to identify and recruit two rural Nebraska communities for Wave 1 (2018–2020), which were randomized to receive either a system for improvement based on a community-driven development model (Wellscapes) or a control standard practice community-driven development model (Collective Impact [CI]) [25]. As part of the system condition of intervention, the community received Wellscapes, which included the following essential elements: (1) a *Community Hub* comprised of community stakeholders and group leaders representing school, afterschool, youth sport, and youth club settings within the community that provided multi-sectorial organization collaboration and knowledge exchange functions [25]; (2) a *Community Action Process*, defined as the four-phase population health quality improvement cycle process – IDPR; (3) a *Multilevel Data Monitoring and Feedback System* consisting of a community population PA outcome monitoring system [26], a setting-level PA monitoring system [27], and data feedback. In contrast, the standard health practice group received the CI protocol to: (1) develop a multi-sectoral organization collaboration and (2) implement standard evidence-based practices targeting moderate-to-vigorous physical activity (MVPA) of children. The focus of this paper is to describe the development and implementation of the *Community Action Process* (*Wellscapes Essential Element #2*) as part of Year 1 of the Wellscapes project. A comparison between the intervention and standard practice conditions is beyond the scope of this paper. The University of Nebraska Medical Center Institutional Review Board approved study procedures (IRB #446-18-EP, IRB #439-18-EX).

### Development of IDPR

Prior to implementation, the research team worked together between January 2018 and August 2018 to develop the IDPR approach underlying the *Community Action Process*. The team relied on our previous population health improvement efforts [21–24] the guiding principles of control systems theory [11, 12], and the application of those principles in within organization quality improvement processes [15, 18], to guide content creation and ensure that IDPR functions as a process that (1) is iterative, (2) incorporates local data into decision-making, and (3) allows adaptation to dynamic local conditions. The overall premise was that IDPR, as a *Community Action Process*, creates a sustainable system that coordinates across sectors and organizations nested within to bring together community members and resources through a continuous feedback loop using local data to foster continuous learning and design solutions for what to produce and how to produce population health opportunities or services. A *Multilevel* *Data Monitoring and FeedbackSystem* operates in tandem with the *Community Action Process* and can help facilitate discussion throughout the entirety of the process [26–28]. The data collection and feedback system included three key measures, each of which has been further described elsewhere [26–28]: a surveillance system measuring child population PA, a system measuring PA in organized settings for children, and a stakeholder survey measuring community system structures and collaboration processes.

#### Phase 1: Investigate

The Investigate phase acts as the sensor to the community system to understand the state of a system, including what constitutes the system, the structure and functional arrangement of local elements comprising the system at different levels, and the underlying rules that govern the organization of the system. The process of investigating uses both quantitative and qualitative methods to rapidly collect data, which then flows to local decision-makers to use during the Design, Practice, and Reflect phases. Local system elements can be grouped into three types of information: (1) geographical and temporal boundaries of the system, (2) drivers of decisions and network of decisions, which work in tandem to elucidate the underlying social structure and governance system makers, and (3) assets or capitals of the system [29–32].

The Investigate workshop consists of three separate activities to guide the *Community Hub* through an investigation of their community to gather local data. The investigation is supported by locally sourced data from the *Multilevel* *Data Monitoring and Feedback System*. To elicit discussion, Hub members answer the overarching question, *“What is our community wellness landscape?”* The first activity centers on understanding the geographical boundary of the system and the settings available within the system and is an important first step in the Investigate phase. A community is characterized as a patch-work quilt or mosaic of behavior settings [33]. Often, the geographical boundary of a community is defined by a pre-determined, standardized boundary such as a zip code or county line [34, 35]. Facilitators guide stakeholders through a place-based mapping exercise to define the geographical space in which the community operates and determine settings in which the community engages.

Facilitators then lead the group through an activity to understand the resources or assets within the local community. Research has shown that understanding the local assets of a system is critical in designing an effective strategy for systems improvement [30, 36]. The Community Capitals Framework (CCF) provides a method for identifying seven different types of assets or capitals, including natural, cultural, human, social, political, financial, and built, that exist in a system [30]. Locally driven mapping of the capitals contributes to understanding the underlying rules of the social system, which allows for decisions to be based on reproducible structures and functions of the local system of producing opportunities.

In the final activity, Hub members complete a social and power network mapping exercise to understand the decision-makers controlling resource allocation for the provision and production of services within the community as a whole and in the identified settings. Although standardized geographical boundaries may be the quicker solution to defining a population, we can also define a population by understanding the dynamic, complex microsystems of self-organized groups (e.g., healthcare providers, not-for-profit community organizations, for-profit firms, local government agencies) of interacting individuals within the geographic area. These interdependent organizations and settings nested within larger whole communities create microsystems of different influence where the decisions, interactions, and feedback loops among individuals and organizations within and across systems are what drive whole-of-community population health outcomes [20, 29, 31, 37].

#### Phase 2: Design

The Design phase builds from Investigate by combining the information gathered during the previous phase to design local solutions. Within the CCF asset development model, activities are designed to (1) frame the problem, (2) ideate solutions, and (3) design a prototype for rapid implementation [38–40]. The process of designing allows for solutions that can be coordinated within and across the levels of the system to create systemic changes to improve population outcomes. In other words, through this process, the Hub decides what to do (provision decision) and how to organize the work (production decision) [41].

The Design phase employs four interrelated activities and uses the data from the Investigate phase provided in a Community Data Report (e.g., place-based map and stakeholder network map) to answer the question, *“How might we strengthen the community wellness landscape?”* Based on collectively reviewing the data, the Hub makes the provision decision through a community headline that is then used to guide their work. Hub members consider how this headline compares to one that would have been written 10 to 50 years ago and what they would like their headline to be in the next five years. The third activity is divided into two parts to encourage further discussion on local community assets and includes 1) brainstorming additional assets of the community using the CCF and 2) listing assets associated with the different settings or places within the community identified by the Hub. The fourth activity moves the Hub to ideation, in which members brainstorm how to move the community toward the ‘headline’ by providing a service(s) (e.g., creating new organized group opportunities). Finally, from the list of ideas generated, the Hub members are provided the autonomy to select at least one idea (with the option of multiple ideas) to move into developing a prototype using a vision board exercise, in which the Hub visually represent the idea(s) using a collection of images, phrases, and materials. Since multiple ideas can be selected at once, consensus across the group is not the goal; rather, it is to have at least one idea to be rapidly practiced in the local community.

#### Phase 3: Practice

The Practice phase of the model puts the prototype into action by asking the question, *“How will we pilot the group opportunity?”* For the purposes of answering the question, the ‘group opportunity’ is the developed prototype determined during the Design phase. The objective of the Practice phase is to expand upon Design activities and move the Hub to rapidly pilot at least one idea or prototype in the community. Rapid implementation of the prototyped idea or solution provides the opportunity to see change in action and make adaptations as needed [15, 18, 21]. This phase first engages Hub members in an activity to develop a Road Map describing strategies for implementing the prototype. The Road Map was not designed as an accountability strategy; instead, it was an overall strategy for organizing efforts around an improved wellness landscape of PA opportunities for children. During the activity, Hub members complete a Road Map worksheet to (1) describe action steps that need to be taken, (2) describe resources needed for the action (e.g., people, places, things), (3) delineate roles, and (4) provide milestones for each action (i.e., timeframe). While developing the Road Map, Hub members are prompted to consider assets already in existence in the community overall and within the Hub. After completion of the workshop, the Hub puts the Road Map components into motion to implement the opportunity.

#### Phase 4: Reflect

Finally, the Reflect phase allows communities to obtain feedback and analyze and reflect on data collected throughout the Investigate, Design, and Practice phases to assess implementation of the prototype. To guide reflection, Hub members are asked the overarching questions, *“How did we improve our community; what was successful and what needs to change?”* and encouraged to consider whether the prototype moved the community toward the headline as well as what adaptations need to be made to the implemented prototype or whether the Hub needs to pivot to another idea to reach population-level health outcomes. While the Reflect phase is the final step or function in the process, in practice, it can be embedded within each component to allow for the flow of information from one process to inform the other processes, creating a continuous feedback loop that allows for adaptations throughout the process.

### IDPR Implementation

After determining the overarching structure and functions of the IDPR process, the research team operationalized these principles into action through a series of four workshops, with each two-hour workshop consisting of questions and activities to guide the Hub through the IDPR process and move the community toward a population health outcome (Table 2). For the purposes of the Wellscapes project, it should be noted that the population health outcome of interest was improved children’s PA through the provision of PA opportunities or services, and while the description to follow focuses on PA, IDPR materials can be adapted to other identified priority areas. Materials were created for each workshop by the research team, which consisted of a facilitator guide, a PowerPoint presentation, and miscellaneous materials to support each workshop activity, such as worksheets.

**Table 2:** Operationalization of IDPR *Community Action Process* in Wellscapes Wave 1, Year 1 (August 2018 – May 2019)

Phase	Theoretical Definition	Implementation	Goal	Guiding Questions	Activity
Investigate	Sensor: Used to detect and measure some effect and feedback the information into the system.	The initial phase of the IDPR process is used to build the capacity of the community to gather and analyze locally sourced data such as community assets and health outcomes at different levels within the community (e.g., community, organization, behavior setting).	To collectively establish a population health goal and understand the community strengths that can be built upon to achieve that goal. To achieve community shared measures around a particular topic.	Where are the places that children and families go in our community?	Place-based map
				Why do children and families go to these places?	Assets map using Community Capitals framework
				Who can make decisions about places for children and families to go in our community?	Network map
Design	Controller: Used to provide decisions and control commands.	The second phase of the IDPR process is used to build the capacity of the group to define the wellness landscape geosocial units and form group consensus as to a potential idea/prototype of a service that can quickly be piloted within the community and assessed for feasibility/viability.	To interpret local data gathered during the Investigate phase and make decisions on a broad vision for the community population health system, as well as decisions on what to produce and how to produce a system of services to reach that vision.	Why do children and families go to identified places in the community?	Community Hub Capitals Worksheet, Questions and Diagrams
				How do we design a wellness landscape of group opportunities for all children and families?	Vision Board
				What organizations, leaders, and community members should be part of our design?	
				How might we strengthen the community wellness landscape?	
Practice	Effector: Used to perform control commands to affect the community system.	Third phase of the IDPR process that allows the community to quickly implement a vision and understand feasibility and viability of the idea/project for the community.	To pilot the idea/prototype of a service group opportunity within the community that was created during the Design phase.	What is the group opportunity?	Road Map
				What is the strategy for implementing the group opportunity in the community?	Mapping individual strengths and roles/responsibilities
				How does the strategy contribute to the community’s wellness landscape?	
				What strengths or capitals are already in place within the community that can be built upon to implement the group opportunity?	
Reflect	Feedback: Used to provide insights into system and push the system away from homeostasis.	Phase of the IDPR process that can occur at the end of the IDP cycle or within each IDP phase. Opportunity to assess what has occurred and create a feedback loop to re-design or enhance.	To evaluate the group opportunity, understand additional information needed by the Community Hub, and determine how to proceed forward in creating a community wellness landscape.	How did the implementation (practice) of the group opportunity go?	Invisible Ruler Activity
				How confident is the group that this opportunity will occur again?	Discussion
				What does this opportunity look like moving forward?	
				How do we build on our individual roles and responsibilities?	
				What are the strengths and weaknesses of the group opportunity?	
				What are the next steps the group needs to take to create a community wellness landscape?	
				What additional information do we need to re-design or enhance the group opportunity?	

The research team worked to build the capacity of the local health department and to identify a local coordinator to plan the logistics of each workshop. The local coordinator was also tasked with recruiting community members to join the *Community Hub* and participate in the workshops, as well as facilitating the workshops. In addition to the workshops, the research team provided technical assistance through monthly meetings via Zoom with the local coordinator.

### Evaluation

Workshops were video recorded using iPads and Swivl^®^, with stakeholders providing consent using a standardized form. Video recordings were saved to a secure location accessible by research staff. Workshop notes were summarized during the workshop and from the video recordings. Attendance was tracked by the research team and the local coordinator, and information gathered during each workshop was reviewed by the research team between workshops.

## Results

The Hub followed the IDPR *Community Action Process* to engage community stakeholders in investigating the local community, which resulted in the rapid development and piloting of at least one prototype group PA opportunity to engage children across the whole community – an afterschool Play-in-the-Park (Table 3).

**Table 3:** Results of IDPR *Community Action Process* in Wellscapes Wave 1, Year 1 (August 2018 – May 2019)

Date	Workshop or Monthly TA Call	# of attendees	# of Organizations Represented	Activities	Deliverable(s)
August	TA Call with LHD	3	2	• Discussion of Grant Materials	
September	Workshop 1: Investigate	7	6	• Place-Based Mapping • Asset Mapping • Community Stakeholder Mapping	• 26 places identified for children and families to engage in PA • Community Stakeholder Map with 11 identified groups
	TA Call with LHD	2	2	• Discussion of Grant Materials	
October	TA Call with LHD	2	2	• Discussion of Grant Materials	
November	Workshop 2: Design	7	5	• Community Capitals Worksheet, Questions and Diagram • Headline Activity • Vision Board Activity	• Community Capitals Map • 3 headlines
	TA Call with LHD	2	1	• Discussion of Grant Materials	
December	TA Call with LHD	3	1	• Discussion of Grant Materials	
February	Workshop 3: Practice	7	7	• Group Opportunity Prototype Brainstorm and Voting Discussion • Group Opportunity Road Map	• Road Map for Prototype: Afterschool Play-in-the-Park
	TA Call with LHD	3	1	• Discussion of Grant Materials	
March	TA Call with Hub	5	4	• Continuation of Workshop 3 • Group Opportunity Road Map for Prototype: Afterschool Play-in-the-Park activity	• Road Map for Prototype: Afterschool Play-in-the-Park
April	Workshop 4: Reflect	7	4	• Invisible Ruler • Determine Next Iteration of Group Opportunity #1 • Investigate-Design for Group Opportunity #2	• Road Map Refined for Prototype: Afterschool Play-in-the-Park
	TA Call with Hub	7	5	• Continuation of Workshop 4 • Refined Group Opportunity Road Map	
May	TA Call with Hub	5	4	• Continuation of Workshop 3 • Group Opportunity Road Map for Prototype: Afterschool Play-in-the-Park activity	• Road Map Refined for Prototype: Afterschool Play-in-the-Park

Notes: TA = Technical Assistance; LHD = Local Health Department; PA = Physical Activity

Prior to the start of the workshops, the local coordinator recruited local community members to participate in the Hub and workshop attendance remained steady throughout the year, with seven community stakeholders present at each workshop. Representation of different community organizations varied throughout the year and included the local health department, local public schools, county extension office, churches, the city mayor’s office, and the parks and recreation department. Four workshops were held between Fall 2018 and Spring 2019, with each workshop lasting approximately two-hours in length. Each workshop was held at a local community organization’s meeting space selected by the local coordinator and lunch was provided to Hub members.

In Workshop 1, *Investigate* (September 2018), the Hub engaged in place-based mapping, community capital/asset mapping, and community stakeholder mapping. Hub members identified 26 places across the community where children and families could go for PA and described these places as being “low-cost or free,” “fun,” in a good “location,” and as a place where children could be with their “friends.” The community stakeholder map identified 11 main groups of community stakeholders as local decision-makers around where children and families go in the community, including clubs, parents, afterschool programs, city council, sporting clubs, chamber, public health department, health clinics, schools, parks and recreation department, and churches.

In Workshop 2, *Design* (November 2018), facilitators utilized the Community Data Report, which included data generated from Workshop 1 (i.e., network map, place-based map, and assets map), to further Investigate the community system and move stakeholders through the Design phase. Using these materials, the Hub brainstormed three headlines to capture the vision of the Hub’s work around child population health PA, including: “[Community name] increases physical activity to make the next generation healthier;” “[Community name] promotes physical activity of youth;” “Creating healthy habits through physical activity.” Determining the second headline would guide the work, Hub members created a vision board to capture potential ideas to implement in the community. Several ideas were discussed, including bringing in someone to coach potential parent volunteers/coaches for youth sport opportunities, providing an instructional experience for kids to learn more skills for a specific sport, maximizing current physical spaces in the community to promote PA, creating intergenerational activities, and determining how to better use public space. Despite generating several ideas, the workshop ended before the group selected an idea to prototype. As a result, prior to Workshop 3 (February 2019), an email was sent to stakeholders to think about (1) the three headlines developed by the Hub and (2) the brainstormed group opportunities discussed as part of the vision board activity in Workshop 2. Hub members were also encouraged to be prepared and ready to engage in the third workshop.

During the third workshop, *Practice* (February 2019), facilitators introduced the goal of the workshop: “Determine a Group Opportunity/Opportunities (Design) that inserts Physical Activity across [the community] and the Strategy (Practice) to do so.” Hub members discussed opportunities to improve childhood PA within the community and added two new ideas to the list from the previous workshop. Hub members narrowed down the eight ideas by voting to focus their efforts on local parks to enhance childhood PA. Due to time constraints, the Hub decided to meet before the fourth workshop to discuss how to further define the group opportunity, ‘parks,’ and how to implement this opportunity. A meeting of Hub members was held over Zoom in March 2019. Before the meeting, Hub members received an email with three questions to guide the online meeting discussion: *“Do you think the group opportunity ‘parks’ is reflective of the data on where kids are going in [the community]?”*; *“From your perspective*, *do parks provide adult-lead opportunity for kids to participate in physical activity?”*; and *“Did you have any ‘ah-ha’ moments after reading through the data provided in the community summary report? Were there at least two data points that seemed interesting to you?”* During the conversation, Hub members decided to implement an afterschool pick-up game in the local park twice per week. A second Hub meeting was held over Zoom in April 2019 to discuss and finalize the Road Map for the group opportunity of offering pick-up games for children in the park after school. Action items were determined along with the resources needed to complete the action idea, the roles of Hub members responsible for the action item(s), and the milestone timeline or dates for when action items needed to be completed. Action items included the creation of a parental consent form, marketing and communication materials, and the purchasing of snacks and equipment. On April 23, 2019, the Hub implemented an afterschool Play-in-the-Park, where children from across the community could come to play and engage in PA.

The final workshop, *Reflect* (April 2019), took place a few days after the initial prototype of Play-in-the-Park was practiced, and feedback regarding previous decisions about what to provide and how to provide it was gathered. The local coordinator from the health department presented data associated with the group opportunity on April 23, 2019, to the Hub. The group opportunity lasted for two hours, and while 20 children were officially signed in for the opportunity, based on observations from the local coordinator, approximately 30–35 children were present in the park and participated in playing the games. Representatives from the local health department were present at the Play-in-the-Park opportunity and brought the equipment and snacks for the children. Three Hub members worked within their networks to determine if volunteers from the local high school and community college could be present for the group opportunity. This networking resulted in two volunteers from the local high school and a representative from the town’s parks and recreation department joining the health department to help supervise the event. During the Reflect workshop, Hub members were guided through an activity known as “invisible ruler,” in which they were asked to answer a series of questions on a scale from 0 to 10 where zero is lowest and 10 is highest. These questions included: *“How confident are you that this group opportunity will take place again?”*; *“How important was the investigate process to making this group opportunity happen?”*; *“How confident are you that the road map and community vision of ‘[Community name] promotes physical activity of youth’ is still the vision you see for your community around youth physical activity?”*; *“How important was the ability to practice this group opportunity?”*; *“What do you think are the successes of this group opportunity?”*. Overall, Hub members reflected that successes of the group opportunity were the location, time of day, length of the opportunity, availability of snacks, parent approval, and Hub member involvement in the opportunity. Hub members decided to implement the park opportunity again in May 2019.

During implementation of the *Community Action Process*, adaptations to the process occurred. For example, while the local coordinator planned the logistics of each workshop, including location and space, snacks, and technology, the research team facilitated the IDPR workshops rather than the local coordinator. In addition to facilitating the workshops in Year 1, the research team provided technical assistance through monthly meetings via Zoom with the local coordinator, however, due to time constraints during the allotted workshops (i.e., two hours), these technical assistance meetings extended to include Hub members beginning in March 2019 to provide Hub members additional time to design and implement a prototype.

## Discussion

The purpose of this paper was to describe the development and components of the IDPR *Community Action Process*. To contextualize IDPR, we used the Wellscapes project, described above, to provide an example of how IDPR can be adapted to guide a community to improve a population health outcome, such as childhood PA. Overall, we found the IDPR *Community Action Process* influenced the *Community Hub’s* design of the provision and production of group opportunities for PA in the community for children. The IDPR process resulted in a rapidly designed and implemented community-driven solution to improve children’s physical activity: an afterschool Play-in-the-Park, where children from across the community could come to play and engage in PA.

The iterative nature of IDPR permits adaptability by stakeholders to organize across sectors and make decisions for and accommodate disruptions that may occur at multiple levels within a community system. For example, there may be organizational-level changes throughout the course of the *Community Action Process*, such as staff turnover within a local organization or implementation of other organizational priorities, creating fluctuations in workshop participation. At the community level, disruptors within the system may include new laws or policies implemented that impact the health outcome being targeted. The adaptability of this *Community Action Process* allows these disruptors to be integrated into the conversation and design of the local solution. Additionally, the rapidness of IDPR enables community members to spend less time in planning stages and instead move through designing and implementation in quick succession to allow for feedback, rapid change, and multiple solutions. As we saw in the Wellscapes project, the Hub designed and implemented a local solution in eight months of the initial meeting (September 2018 – April 2019), while other models, such as CI and Mobilizing for Action through Planning and Partnerships (MAPP), require long planning-only phases as part of the process (approximately 1–2 years) [42, 43]. As a result, solutions designed and implemented may be irrelevant by the time they are implemented because of a local community’s dynamic and rapidly changing landscape. As such, a process like IDPR facilitates the design and implementation of a local solution for improving the targeted health outcome that fits the local community and has a greater chance of success and sustainability.

Additionally, a local data system is imperative for obtaining feedback information and the successful operation of IDPR. Throughout the process, community members can look closely at both local objective data, as well as engage in the generation of data such as local community assets (e.g., community capitals) to help drive decision-making and design local solutions. The integration of a continuous *Data Monitoring and Feedback System* enhances the local community’s ability to design and implement solutions (i.e., opportunities and services) that meet the needs of the local population.

## Conclusion

IDPR is an innovative community-engaged action process to design local solutions that improve population-level health. Current community approaches to improvement, such as Collective Impact [19], rely heavily on quality improvement efforts in which the community is treated similarly to a production line where no preexisting conditions exist. In CI, the coalition defines the vision (system goal), forms a plan to reach the system goal, implements the plan, and holds stakeholders accountable to the plan. The plan often involves implementing evidence-based practices to improve the community and reach the system goal. The underlying assumption with models such as CI is that the work is being conducted within a homogenous social system, and accountability and evidence-based practices are the key to ‘fixing’ the community. However, in implementation, this solution falls short because there is no one-size-fits-all solution. Community stakeholders are not members of a production line within a homogeneous hierarchical organizational social system; community stakeholders are complex, autonomous, and dynamic, which creates an immense amount of complexity and variability that requires a different process of coordination. In comparison, IDPR is based on a dynamic control systems theory approach utilizing feedback control system principles that embraces the reality that community-level health improvement requires a flexible, iterative process. This process accounts for the multiple heterogeneous, autonomous units within the community and offers a learning environment where information can be exchanged between units. It also uses an asset and strengths-focused approach to investigate the community. The phases of IDPR present this opportunity for communities to engage in defining what is the “right” thing locally by investigating their local community system, designing appropriate solutions for units to implement within the community’s local system, and then running continuous local experiments (practice, reflect) to allow communities to adapt to changing local conditions and make decisions on how best to proceed.

## Data Availability

The datasets generated and analyzed during the current study are not publicly available. Following NIH and IRB data sharing policies, de-identified data will be made available after publication of the main findings of R01CA215420 from the PI David A. Dzewaltowski, PhD.
